# Supplementary material for: Effects of Fructose and Palmitic Acid on Gene Expression in Drosophila melanogaster Larvae: Implications for Neurodegenerative Diseases
Source: Int J Mol Sci. 2023 Jun 17;24(12):10279. doi: 10.3390/ijms241210279 (PMC10299333; doi:10.3390/ijms241210279)
Supplement: Supplementary file 1 [file ijms-24-10279-s001.zip › ijms-2402043-supplementary.pdf]

Supplementary Table S1. Orthologous genes in humans with differential expression. The table shows the orthologous genes in humans which are described throughout the text and are described in the figures that showed differential expression in the diet supplemented with palmitic acid and fructose (MD) with respect to the control diet (ND).

**Downregulated Genes**		**Upregulated Genes**	
**EC 4.1.1.15**	Glutamic acid decarboxylase	**EC 7.1.1.2**	NADH:ubiquinone reductase
**EC 6.3.1.2**	Glutamine synthase	**EC 1.6.99.3**	NADH dehydrogenase
**EC 2.4.2.14**	Amidophosphoribosyltrans-ferase	**EC 7.1.1.8**	Quinol-cytochrome-c reductase
**EC 1.14.16.2**	Tyrosine hydroxylase	**EC 1.9.3.1**	Cytochrome-c oxidase
**EC 53.3.12**	Dopachrome tautomerase	**EC 7.1.2.2**	F1-ATPase
